# The Book World of Medicine and Science

**Published:** 1911-12-02

**Authors:** 


### The Book World of Medicine and Science.

### THE PUBLIC AND THE DOCTOR.

Medical Science of To-day. By Willmott Evans, M.D., F.R.C.S. (London: Seeley, Service and Co., Ltd., 38 Great Russell Street. "Science of To-day" Series. Price 5s. net.)

We congratulate alike Mr. Willmott Evans and Messrs. Seeley and Co. on their respective shares in the making of this book. The former has had the courage to risk the dissent of his professional colleagues—some of whom will doubtless think it *infra dig.* for a consultant to talk, in this cheerful, simple manner, to the public at large about the work that he does and the advances that his art is daily making; the latter have had the courage to publish an original book which, if certain professional colleagues, again, are to be believed, will not sell, since the public does not care to know about such things. Great is Diana of the Ephesians—and great, apparently, according to those whose most earnest desire it is to introduce what some people would call "syndicalism" into medicine, is the confidence of the public in the profession. We have always insisted that it would be well if the profession took the public into its confidence in a somewhat more detailed fashion than it has hitherto done. Nevertheless, few medical men have deemed the matter of sufficient interest or importance to be dealt with in a serious spirit. Mr. Evans, whose reputation as a surgeon gives him an unquestioned authority, has struck out a new line, one which we hope will be followed by other members of the profession, for such books as this can only be productive of good.

Let us briefly summarise his work. In thirty-one chapters, every one written in a simple, attractive style which avoids undue technicalities without investing the subject with a commonplace morbidity of sentiment—the prevailing error in American works of this genre—he traces the development and progress of modern medicine. A lay reader who takes up this book can, by perusing it, obtain a succinct and comprehensive idea of what is nowadays embraced under the term medicine; he can, almost at a glance, see the difficulties with which his doctor has to contend. Similarly he can form certain conclusions, tending to show that there has been a decided advance, conclusions which must inevitably induce him to view with hope the struggles which still lie in front of the profession in its fight against disease. Such a book, then, is of the greatest value to the layman; its value to the profession is not less, for it will be of decided help to the doctor in showing the general public what are the problems of modern medicine, how they are being attacked, and in what manner they are being overcome.

The book is well illustrated, on the whole, the skiagrams in particular having been chosen with great care; every illustration is really elucidative. Mr. Evans is generally accurate in his statements, except, perhaps, on points of history; but medical history is such a neglected subject that the few slips made are excusable. For all that, we should like him, in the next edition (which, we trust, will not be long delayed) to pay the deserved tribute that every surgeon owes to Semmelweis. In the chapter dealing with what modern surgery can do, he might also with advantage allude to the striking results obtained by Gluck and others in the surgery of lung cases, and by Spitzy in nerve anastomosis in cases of spastic diplegia, while a note on the improvements in eye surgery might also be of interest. Among the industrial diseases sulphur poisoning and chronic permanganate poisoning should have a place. In the chapter on diagnosis, modern methods of examining the stomach and intestines, the bronchoscope, the œsophagoscope, and the cystoscope should be explained. But he has managed to cram so much within the limits at his command that any criticism of omissions must be cavilling. As the work stands, it amply fulfils the promise held out on the title-page, "A popular account of the more recent developments in medicine and surgery." We sincerely trust that practitioners will not only buy the book themselves—for it deserves a permanent place on their shelves—but bring it to the notice of their patients, and thereby contribute to the success of the propaganda of public enlightenment.

---

National Conference on the Prevention of Destitution. Report of Proceedings of the Conference held at Caxton Hall in May and June 1911. (London: P. S. King and Son. Price 10s. 6d. net.)

Contains the full report of the addresses and papers delivered and read at the Conference. Many of them are of great and lasting interest; others, again, have a more ephemeral value. A book which everyone interested in such questions as education, employment, physical deterioration, and social problems generally ought to read and to give a place on his shelves.

Sir John Burdon Sanderson. A Memoir by the late Lady Burdon Sanderson. With selections from his papers and addresses. (Oxford: The Clarendon Press.)

This is a record of Sir John Sanderson's life and work, illustrated with some interesting photographs and containing several abstracts of his speeches and addresses on questions which appear to have a more or less permanent interest. The address on "The Consumptive Bread Earner," for instance, deserves the widest publicity that can be given it. Admirers of the late Regius Professor of Medicine's work will find this memorial a welcome addition to their libraries. For the general reader it contains little that is of outstanding interest.

Diseases of the Skin. By Sir Malcolm Morris, K.C.V.O. Fifth edition, revised by the Author with the assistance of S. Ernest Dore, M.D., M.R.C.P. (London: Cassell and Co., Ltd. 1911. Price 10s. 6d. net.)

A text-book so well known and so deservedly popular as this needs no introduction to the profession. The new edition has been revised and brought up to date by the inclusion of much new matter. A very interesting section is that which deals with modern research work in syphilology. The author accepts the positive Wasserman reaction as a conclusive test of syphilis when leprosy and sleeping sickness can be ruled out. This seems to us a very optimistic dictum, since it has been shown that the reaction is positive in a great many cases in which no evidence of syphilis can be obtained. For instance, it has been shown that the blood of patients anæsthetised with combined scopomorphine methods give the reaction. Much more cautious is the opinion expressed with regard to the salvarsan and hectine methods of treatment, though the author states that "606" is to be regarded as a remedy which will hold "a prominent and enduring place in the treatment of syphilis." The illustrations are beautifully executed and add much to the value of the book, which may honestly claim to be the best short manual on the subject with which it deals that the English-speaking practitioner can buy.

